# The Building Healthy Eating and Self-Esteem Together for University Students Mobile App to Treat Eating Disorders: User-Centered Research Design and Feasibility Study

**DOI:** 10.2196/43504

**Published:** 2023-07-12

**Authors:** Kelsie T Forbush, Kara A Christensen Pacella, Marianna L Thomeczek, Sara R Gould, Danielle A N Chapa, Brianne N Richson, Victoria L Perko, Joseph Ayres, Yiyang Chen, Sonakshi Negi

**Affiliations:** 1 Department of Psychology, University of Kansas Lawrence, KS United States; 2 Department of Psychology, University of Nevada, Las Vegas Las Vegas, NV United States; 3 Department of Developmental and Behavioral Health Children's Mercy Kansas City Kansas City, KS United States; 4 ReefPoint Group, LLC Tysons, VA United States

**Keywords:** Building Healthy Eating and Self-Esteem Together for University Students, BEST-U, eating disorders, EDs, college, university, students, cognitive behavioral therapy, dialectical behavioral therapy, mobile health, mHealth, mental health, guided self-help, therapy, app, treatment, design, usability, testing, development, development, acceptability, cognitive, mobile phone

## Abstract

**Background:**

University students are an at-risk group for the development of eating disorders (EDs); however, many college campuses lack sufficient resources to provide ED specialty care. Students report unique reasons for not seeking ED treatment, including the desire to solve the problem on their own (eg, seeking help from friends, self-medicating, or waiting to see if their problems improve), inability to afford treatment, lack of time to participate in the treatment, fear of seeing their primary care physician, and lack of recognition of their issues as an ED. Mobile health (mHealth) apps may be a cost-effective, helpful adjunctive tool to overcome personal and systemic barriers and encourage help seeking.

**Objective:**

This paper describes the development, usability, and acceptability of the Building Healthy Eating and Self-Esteem Together for University Students (BEST-U) mHealth smartphone app, which is designed to fill critical gaps in access to ED treatment on college campuses.

**Methods:**

We undertook a 4-phase iterative development process that focused on user-centered design. The 4 phases included needs assessment based on literature reviews, prototype development and initial evaluation in a pilot trial, redesign, and further pilot-testing to assess the usability and acceptability of the final version of the mHealth app. Acceptability and user satisfaction were assessed using an ad hoc survey that ranged from 1 (strongly disagree) to 7 (strongly agree).

**Results:**

Our needs assessment identified a lack of accessible and affordable treatments for university students. To help meet this need, the BEST-U prototype was designed as an 11-week program that provided interactive, weekly *modules* that focused on second- and third-wave cognitive behavioral skills. The modules focused on topics such as psychoeducation, reducing thought distortions and body checking, improving body image, interpersonal effectiveness, and behavior chain analysis. The content included interactive quizzes, short answer questions, daily and weekly logs, and surveys completed in the app. BEST-U was paired with brief 25-30 minutes of weekly telehealth *coaching* sessions provided by a licensed provider or supervised trainee. Pilot-testing revealed minor issues with one module of the app content, which some participants viewed as having low relevance to their experience and therapist concerns about the organization of the app content. These issues were addressed through the removal, addition, and reorganization of BEST-U modules, with the help of therapists-in-training across 2 workshops. The revised version of the BEST-U app had a grand mean acceptability rating of 5.73 out of 7. The participants completed 90.1% (694/770) of the BEST-U modules, indicating high compliance.

**Conclusions:**

BEST-U is a new, acceptable, and user-friendly mHealth app to help therapists deliver brief, evidence-based cognitive behavioral interventions. Owing to its acceptability and user-friendly nature, BEST-U has high user compliance and holds promise for future implementation and dissemination in university mental health settings.

## Introduction

### Background

Eating disorders (EDs) are a major public health concern associated with substantial psychiatric and medical morbidity, with one of the highest mortality rates of all mental illnesses [1,2]. Given the negative personal and public health impact associated with EDs, it is important to identify and treat them early to prevent long-term health problems. In community-based samples, EDs occur in 13% to 18% of young women and 3% to 5% of young men [3,4]. University students are at particularly high risk for the development of disordered eating behaviors, such as extreme dieting or fasting, binge eating, and purging [5]. For example, initial findings from the Healthy Minds Study—an annual web-based survey of students (that includes traditional and nontraditional students) at more than 180 US universities—indicated that the prevalence of EDs ranged from 13.5% in women to 3.6% in men [6]. Other studies have shown that rates of unhealthy weight-control behaviors and EDs have significantly increased among college students since the mid-1990s [7]. Recent data from the Healthy Minds Study have shown that depending on the screening measure used [8-10], the prevalence of EDs in university campuses ranges from 11.9% to 40.2%, suggesting that there is a critical need for quality ED treatment services in university campuses.

Despite the relatively high prevalence of unhealthy weight-control behaviors and EDs among university students, only 20% to 21.9% of these students reported that they had received treatment for their eating concerns [6,11]. Reasons for avoidance to seek care included a desire to solve the problem on their own (eg, seeking help from friends, self-medication, or waiting to see if their problems improved), inability to afford treatment, lack of time to participate in treatment, and fear of seeing their primary care physician [11]. Tavolacci et al [11] postulated that the fear of seeing one’s physician was likely explained by experiencing guilt or shame related to discussing binge-eating behaviors; thus, self-stigma may present another barrier to treatment seeking in college students with an ED. In addition to these barriers, research suggests that only approximately half of university women with an ED recognize that they have a problem [12], indicating that low mental health literacy may prevent students with an ED from seeking care.

Several apps have been developed to address the needs of people with EDs, including Guided Self-Help Cognitive Behavioral Therapy (CBT-gsh)+Noom Monitor, Recovery Record, Break Binge Eating, and Student Bodies–ED (SB-ED). All of these programs have demonstrated preliminary efficacy in reducing ED pathology [13-16]; however, there are a few features of each program that could impact treatment engagement, including the use of the app for monitoring rather than as a tool for intervention delivery and limited use of therapist or coaching resources to potentially increase treatment adherence. For example, studies of the Noom Monitor used the app to replace the paper-and-pencil monitoring logs of meals, ED behaviors, and weight, methods which are traditionally used in therapist-supported CBT-gsh. Thus, clients in the CBT-gsh+Noom monitor study were required to refer to the *Overcoming Binge Eating* book to access intervention materials [13,17]. Studies using the Recovery Record app followed the format of entirely self-guided Cognitive Behavioral Therapy (CBT; ie, no coaching) [15] or were designed for people stepping down from more intensive treatment for anorexia nervosa and thus, have not been tested in people with binge-spectrum EDs [18]. Although effective in reducing global ED symptoms, the *Break Binge Eating* app was entirely self-guided [14]. Finally, the SB-ED app has also shown promising results in improving ED symptoms [16]. However, the SB-ED app delivered coaching via SMS text message and required students to complete forty 10-minute sessions over 6 months, and therefore, the engagement was lower than ideal (eg, “overall engagement with the intervention was 31%”), which was defined as the percentage of content completed [16]. SB-ED was also designed for women, which means it may not fully address the needs of men with ED symptoms.

### Objectives

Each of the existing mobile health (mHealth) apps for the treatment of EDs has the potential to expand access to ED care and has several notable strengths. However, to best serve the diverse range of clients who experience EDs, it is necessary to have a variety of treatment options that match various clients’ needs. Through a review of the existing mHealth apps for EDs, it is clear that there is room for improvement. Additional mHealth apps are needed that provide treatment that is less intensive than traditional outpatient CBT therapy but with more therapist involvement than solely app-based interventions. With this in mind, our treatment team created the Building Healthy Eating and Self-Esteem Together for University Students (BEST-U) mHealth app. This paper describes the process of developing and pilot-testing BEST-U for feasibility and user acceptability. BEST-U is a brief, guided self-help mHealth app that is based on second- and third-wave cognitive behavioral therapies. Second-wave therapies include traditional cognitive behavioral techniques, such as identifying and challenging distorted cognitions and behavioral activation. Third-wave therapies often include elements of second-wave therapies but with an enhanced focus on acceptance, mindfulness, cognitive defusion, dialectics, values, interpersonal relationships, and the use of experiential techniques [19]. BEST-U was developed for iPhone and Android on the PiLR MEI Research platform [20] and was designed for non–low-weight university students of all genders, as well as both traditional and nontraditional students. In the *Methods* section, we describe the 4-phase iterative user-centered process that we undertook to create BEST-U, which included a needs assessment, initial development, refinement, and pilot-testing of usability and acceptability.

## Methods

### Overview

This paper describes the development of the 4-phase BEST-U app iterative user-centered process that involved (1) a needs assessment based on literature reviews, community treatment, and provider surveys; (2) prototype development and initial evaluation in a pilot trial with stakeholder and therapist inputs; (3) redesign; and (4) further pilot-testing to assess the usability and acceptability of the final version of the mHealth app.

### Ethical Considerations, Informed Consent, and Participation

This study was approved by the University of Kansas institutional review board (#STUDY00144380). Participants provided signed informed consent before engaging in any study-related procedures. Participants also completed release of information forms so that their medical evaluation results could be shared with the study team. All data were kept confidential, and identifying data were maintained in locked files or kept in our password-protected dual-authenticated research drive. Intake and progress notes were maintained within Healthie, which is a password-protected, Health Insurance Portability and Accountability Act (HIPAA)–compliant web-based electronic health record platform. The participants earned US $50 for the initial 2-hour intake assessment and medical assessment. Next, they received US $5 per week for completing the modules within the mobile app (US $50). After the treatment ended, they received US $5 for the end-of-study survey, US $10 for the 3-month survey, and US $10 for the 6-month survey. Participants received an additional bonus of US $25 if they completed all modules and the follow-up surveys.

### Phase 1: Needs Assessment

Our needs assessment included 2 components. First, we surveyed the directors of treatment centers on our local campus (n=3) and the state in which our university was located (n=5) to assess the availability of ED treatment services and relevant information, such as waitlist times and whether the provider took insurance. Our provider survey was administered in 2018 and was used to inform our initial treatment design. Second, we conducted a needs assessment based on a literature review to inform our selection of treatment modality.

### Phase 2: Prototype Development and Initial Evaluation in a Pilot Trial

The initial version of BEST-U, which we called BEST-U 1.0, was developed over a 3-month period based on a collaboration between 2 clinical psychologists (KTF and SRG), one of whom worked primarily as an academic researcher and the other as a clinician with children and adolescents in an academic medical center. During this 3-month period, the study clinical research coordinator met once per week with the treatment development team to discuss content and create a paper-based outline of session content that was reviewed and revised at subsequent meetings. The clinical research coordinator also met, as needed, with the study principal investigator for informal check-ins, progress reports, and to ask questions. Once an outline was approved by all team members, the content was programmed into the PiLR MEI Research platform and pilot-tested by trainees to ensure an attractive appearance and accurate collection of data. After the initial creation of BEST-U 1.0, we sent a large-scale web-based REDCap (Research Electronic Data Capture; Vanderbilt University) survey to all enrolled students (approximately 28,500) at a large midwestern university to screen for EDs and recruit participants in the initial clinical trial. The enrolled students were eligible to participate in the survey if they were aged >18 years. Students were encouraged to participate in the screening survey, regardless of whether they had eating or body image concerns.

**Figure 1:**
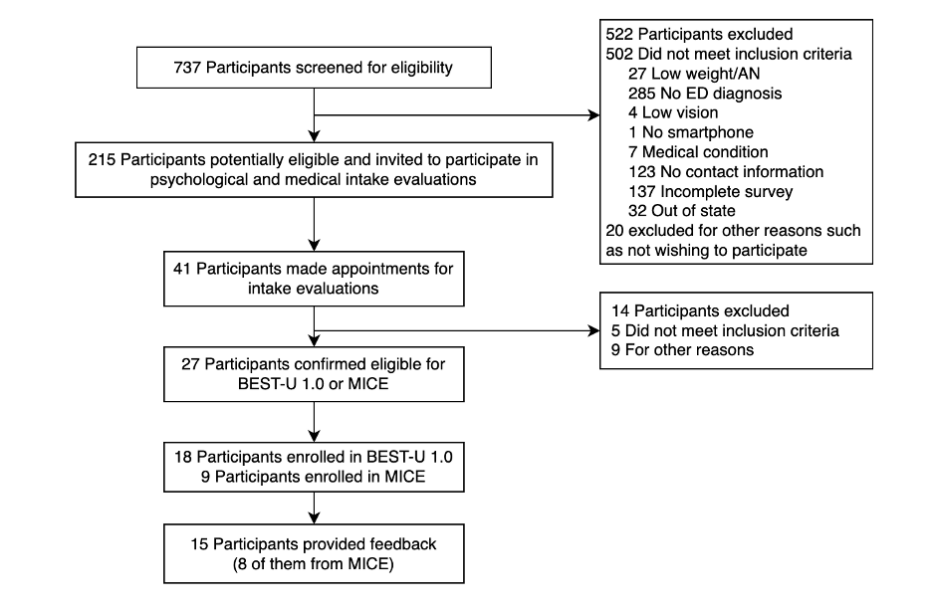
CONSORT (Consolidated Standards of Reporting Trials) diagram of Building Healthy Eating and Self-Esteem Together for University Students (BEST-U) 1.0. AN: anorexia nervosa; ED: eating disorder; MICE: Multiple Baseline Design for Improving College-Student Eating.

A total of 737 students completed the initial web screening, of which 215 (29.2%) were potentially eligible based on their web survey responses. Participants were potentially eligible if they reported disordered eating symptoms that aligned with a potential ED diagnosis, had access to a smartphone, and lived in a state in which psychologists on our team were licensed. Participants were excluded if they had a BMI <19, reported uncorrected vision problems that could prevent their use of a smartphone, or reported a medical circumstance that could interfere with weight or appetite (eg, pregnancy, diabetes, neurological condition). Potentially eligible participants were invited to complete psychological and medical intake evaluations. A total of 41 individuals completed both intake evaluations. After the medical and psychological intake evaluations, 27 participants were deemed eligible and 18 were enrolled in the trial. Notably, at this stage of the trial, the other 9 participants were enrolled into a separate multiple-baseline single-case research design protocol, and 8 of these participants completed the protocol, the intervention for which consisted of BEST-U 1.0 [21]. Out of all enrolled participants in BEST-U 1.0 and the multiple-baseline single-case study, 15 participants provided end-of-treatment feedback about treatment satisfaction and app usability (7 from BEST-U and 8 from the multiple-baseline study). See Figure 1 for a CONSORT (Consolidated Standards of Reporting Trials) diagram of recruitment and enrollment flow. Table 1 provides the demographic information (age, gender, and race and ethnicity) of all students who responded to the screening survey and students who enrolled in the BEST-U 1.0 trial.

The participants completed a 17-item ad hoc questionnaire that measured their opinions on app usability, attractiveness, and satisfaction with coaching. The questions were rated on a 7-point Likert scale that ranged from 1 (strongly disagree) to 7 (strongly agree). The questions assessed the extent to which participants felt comfortable using the app, enjoyed participating in the study, felt the amount of time answering questions in the app was reasonable, felt the amount of time reading the content of the modules in the app was reasonable, felt the app was attractive, the length of the overall treatment program, participant perceptions of the extent to which their eating and body image changed, and several questions about their experience with their coach. In addition to the ad hoc questionnaire, participants responded to 2 open-ended questions that asked, “What were the best things about the BEST-U program?” and “What changes do you recommend to the BEST-U program?”

**Table 1:** Demographic characteristics of Building Healthy Eating and Self-Esteem Together for University Students 1.0’s participants.

Characteristics			Screened (n=737)		Enrolled (n=18)
Age (years), mean (SD; range)			21.60 (4.99; 18-78)		23.00 (6.92; 18-45)
**Gender, n (%)**					
	Cisgender men	159 (21.6)		1 (5.6)	
	Cisgender women	536 (72.7)		16 (88.9)	
	Transgender men	5 (0.7)		0 (0)	
	Transgender women	1 (0.1)		0 (0)	
	Gender nonconforming	6 (0.8)		0 (0)	
	Another gender identity	0 (0)		0 (0)	
	Prefer not to answer or missing	30 (4.1)		1 (6)	
**Race and ethnicity, n (%)**					
	Asian	64 (8.7)		1 (5.6)	
	Black	29 (3.9)		1 (5.6)	
	Hispanic	69 (9.4)		2 (11.1)	
	Native American or Pacific Islander	19 (2.6)		0 (0)	
	White	624 (84.7)		16 (88.9)	
	Multiracial	36 (4.9)		0 (0)	
	Other race or not specified	39 (5.3)		0 (0)	

### Phase 3: Redesign

After the initial pilot-testing, we evaluated end user feedback collected in phase 2 and held 2 workshops with trainees (n=4) who served as BEST-U 1.0 treatment providers and made minor changes to the app based on their feedback. Trainees were graduate students enrolled in a Doctor of Philosophy program or postdoctoral fellows who had completed a graduate-level course on the treatment of EDs or the University of Oxford’s web-based training on CBT-Enhanced (CBT-E). We used coach feedback to redesign the app by removing 1 module, adding a new module, and refining 1 module to provide more interactive content. We also used coach feedback to redesign the web dashboard that coaches used to review their clients’ weekly data.

### Phase 4: Further Pilot-Testing of Usability and Acceptability

**Figure 2:**
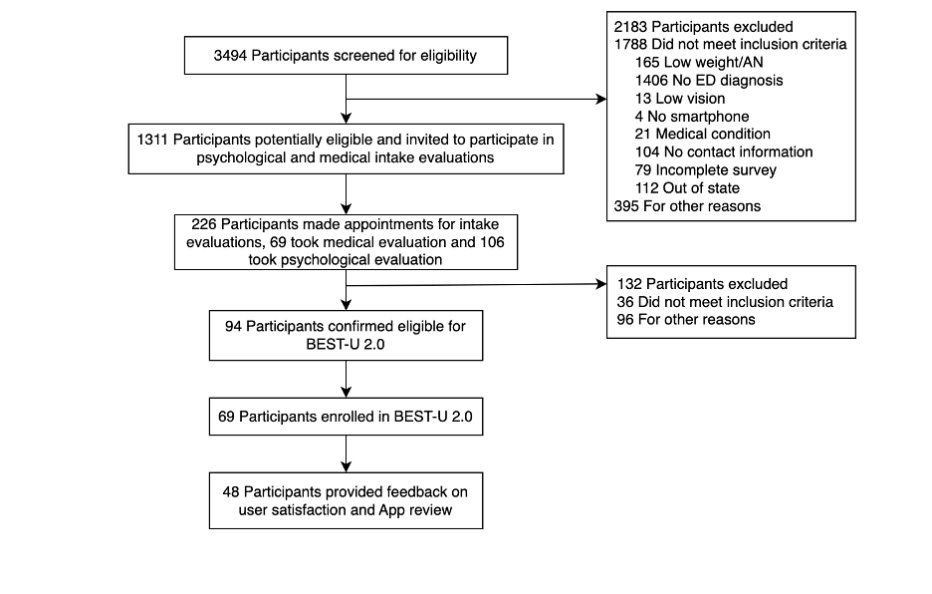
CONSORT (Consolidated Standards of Reporting Trials) diagram for Building Healthy Eating and Self-Esteem Together for University Students (BEST-U) 2.0. AN: anorexia nervosa; ED: eating disorder.

The goal of phase 4 was to test whether the overall treatment remained acceptable after the implementation of minor changes. Thus, we conducted an additional pilot study to assess the usability and acceptability of BEST-U 2.0 in university students (n=47) with a non–low-weight, binge-spectrum ED using the same inclusion and exclusion criteria that we used in phase 2. Overall, 3494 participants completed the initial web screening survey. On the basis of web survey responses, 1311 participants were potentially eligible and invited to complete the psychological and medical intake evaluations. A total of 226 participants were interested in participation and completed the psychological and medical intake evaluations. Notably, recruitment for this phase began during the COVID-19 pandemic, which resulted in some participants delaying or canceling participation because they did not want to complete the in-person medical examination. After the medical and psychological intake evaluation, 94 participants were eligible, and 69 participants were enrolled in the study. Reasons for ineligibility included the need for a higher level of care, the presence of a primary substance or alcohol use disorder, and severe primary co-occurring psychopathology requiring stabilization or intervention. A total of 47 participants provided end-of-treatment feedback on treatment satisfaction and the app. Participants completed the same ad hoc usability and feasibility questionnaire and open-ended survey items that were administered in phase 2. Figure 2 provides a CONSORT diagram of the phase 4 recruitment and enrollment flow. Table 2 shows the demographic information (eg, age, gender, and race and ethnicity) of students who completed the screening survey and the sample of participants who enrolled in BEST-U 2.0.

**Table 2:** Demographic characteristics of Building Healthy Eating and Self-Esteem Together for University Students 2.0’s participants.

Characteristics			Screened (n=3494)		Enrolled (n=69)
Age (years), mean (SD; range)			22.95 (6.63; 18-76)		25.56 (8.01; 16-55)
**Gender, n (%)**					
	Cisgender men	790 (22.6)		12 (17.4)	
	Cisgender women	2326 (66.6)		50 (72.5)	
	Transgender men	26 (0.7)		2 (2.9)	
	Transgender women	3 (0.1)		0 (0)	
	Gender nonconforming	89 (2.6)		2 (2.9)	
	Another gender identity	40 (1.1)		1 (1.4)	
	Prefer not to answer or missing	220 (6.3)		2 (2.9)	
**Race and ethnicity, n (%)**					
	Asian	416 (11.9)		5 (7.2)	
	Black	181 (5.2)		6 (8.7)	
	Hispanic	367 (10.5)		10 (14.5)	
	Native American or Pacific Islander	94 (2.7)		0 (0)	
	White	2804 (80.3)		56 (81.2)	
	Multiracial	202 (5.8)		2 (2.9)	
	Other race or not specified	217 (6.2)		4 (5.8)	

## Results

### Phase 1: Needs Assessment

#### Provider Survey

We surveyed centers located on our campus (n=3) and in the state in which our university is located (n=5). The results of the assessment indicated that 2 campus-based treatment centers did not treat EDs and reported referring students with EDs to the local community. The remaining campus-based treatment center was a graduate training clinic in which Doctor of Philosophy students treated clients under the supervision of a licensed faculty. The training clinic accepted student and nonstudent clients using a sliding-scale fee and did not accept insurance. The waitlist at the training clinic ranged from 1 to 3 months, although the waitlist increased substantially with the onset of the COVID-19 pandemic, with the current therapy waitlist at 3 months and the waitlist for assessments at 12 months.

One state-wide treatment center was a children’s hospital that did not regularly accept new, young-adult clients. The other 4 treatment centers were outpatient ED specialty treatment centers located approximately 45 minutes from the campus. These centers received between 10 and 20 calls per month from new clients seeking services for the treatment of EDs. Two centers had waitlists of 2 and 4 months, respectively, whereas the other 2 centers referred clients to other centers and did not maintain a waitlist. Two centers did not take insurance, whereas the remaining 2 centers took the Blue Cross Blue Shield or both Blue Cross Blue Shield and Tri-CARE insurance, respectively. Only 1 center took Medicaid or Medicare insurance. Two centers saw people without insurance, one center had <5% of clients reporting no insurance and the other center had 21% of uninsured individuals, with some clients opting out of insurance coverage owing to high deductibles for mental health care, and 6% received pro bono services. Regarding the diversity of ED admissions, 1 center did not keep track of the specific numbers of racial, ethnic, or sexual minority clients. However, the remaining 3 centers reported an average of 5% of clients from racial or ethnic minority groups (range 1%-9%). The 3 remaining centers reported an average of 2% of clients with a minoritized sexual orientation (range 0%-5%). None of the providers offered services to non–English-speaking clients.

Overall, the provider survey indicated substantial gaps in outpatient services on campus and in the local community for students with EDs. Two on-campus treatment centers did not accept clients with an ED, and the only campus center that treated EDs had long wait times. The lack of on-campus services was particularly problematic given that *nearby* treatment centers were at a distance of 45 minutes from the campus, requiring students to drive or find transportation to attend in-person therapy.

The results of the provider survey led our treatment development team to consider ways to meet the high demand for ED services at scale. In particular, to address the need for accessible ED treatment in the general population, research has increasingly focused on developing easy-to-disseminate interventions that address barriers to accessing care [22]. For example, CBT-gsh lowers therapist burden and increases scheduling flexibility by providing self-help material, including psychoeducation and cognitive behavioral skills through a textbook with brief *coaching* sessions that last 25 to 30 minutes each week. CBT-gsh has been delivered in a range of doses, from as little as 6 weeks to as much as 7 months, spread across 4 to 40 sessions. The training of the *Coach* or guide varied from graduate or doctoral students to facilitators with no formal training, with 1 study using primary care providers [23,24]. Although most coaching sessions have been delivered via face-to-face sessions, other delivery modalities, either individually or in a group format, include the web, telephone, and SMS text messaging. However, few studies have included telemental health as the primary modality for CBT-gsh coaching. Thus, we conducted an additional needs assessment through literature reviews to evaluate the potential feasibility of creating an mHealth CBT-gsh treatment for university students, which we describe in the next section.

#### Literature Review

A Cochrane review found that CBT-gsh was superior to “pure” self-help and waitlist control and equivalent to traditional CBT for improving ED-specific and general psychopathology [25]. Traviss-Turner et al [23] conducted a review and meta-regression of 30 randomized controlled trials of CBT-gsh and found a moderate effect on total ED symptoms (-0.46) and a small effect on binge abstinence (-0.20). Despite the promising effects of CBT-gsh as a low-intensity efficacious treatment for EDs, Traviss-Turner et al [23] noted three important limitations of CBT-gsh:(1) in some studies, attrition rate was as high as 88%, although the reasons for dropout were not explored in their meta-regression; (2) few studies tested the effectiveness of CBT-gsh in university students; and (3) most studies provided guidance through a textbook, and few programs leveraged advances in technology using eHealth and mHealth as modes of guidance.

Since the publication of the review by Traviss-Turner et al [23], there has been increased interest in using mHealth to address mental health disparities in college students [24,26]. However, within the field of EDs, there is only 1 CBT-gsh mHealth app for college students. Specifically, Fitzsimmons-Craft et al [24] developed and tested an innovative CBT-gsh program, SB-ED, designed to treat college women with EDs. SB-ED consists of 40 lessons that last for 10 minutes delivered through a smartphone app. The lessons focused on psychoeducation, meal planning, tracking and self-monitoring, and strategies from CBT-E for EDs (eg, body image, shape and weight avoidance, challenging negative thoughts, and relapse prevention). The students were matched with a health coach who sent weekly text messages throughout the program to provide ongoing feedback, support, and encouragement. Coaches also provided 2 optional 20-minute phone calls; 1 call was scheduled at the beginning of the program to establish rapport, discuss goals, and address barriers (session 1), and the other phone call was provided at the end of the treatment (session 40) to review the client’s progress and relapse prevention strategies.

Data from a cluster randomized trial of 26 US universities showed that SB-ED was an effective intervention [16]. Participants randomized to the SB-ED (vs treatment as usual) had significant reductions in total ED symptoms that were maintained at follow-up. There were no significant differences between the groups in abstinence from ED behaviors at end-of-treatment or follow-up. However, the SB-ED group had lower frequencies of binge eating and compensatory behavior at the end of treatment; compensatory behavior (but not binge eating) frequency remained significantly lower than that of the control group at follow-up.

Despite the promising data on the usefulness of SB-ED for college students, there were certain limitations that lowered its impact and reach. First, the SB-ED was developed for women with an ED, even though approximately 3% to 5% of people with an ED are boys or men [3], and the rates of EDs in transgender people or nonbinary populations appear to be elevated compared with the general population [27]. Furthermore, randomized controlled trials of SB-ED have been conducted in all-female samples, with students who identified as nonfemale not included in the study [16,28]. SB-ED has been administered among different genders, but neither study has a randomized controlled design. For example, in 1 open trial, the authors did not report results broken down by gender [24], whereas in the other, men or gender-diverse students were offered the use of SB-ED but were not included in the published trial results [29]. Thus, there are currently no CBT-gsh mHealth treatments that have been developed for men or gender-diverse university students, and there is limited research on their efficacy in male-identifying populations. Second, SB-ED provides coaching via SMS text messaging and 40 treatment sessions, which led to only 31% of the content being accessed by participants [16]. Newer models of CBT for nonunderweight EDs indicate that treatment gains can be achieved in as few as 10 sessions, with similar remission rates and improvements in quality of life compared with standard-length CBT [30]. Short-length CBT was also associated with a 15% less dropout rate than standard-length CBT [30], suggesting that clients may have potentially received a higher *dose* of the intervention in the shorter-length version because more individuals accessed the full intervention. Finally, the benefits of short-length CBT were maintained at the 6-month follow-up, suggesting that shorter-length CBT is a cost-effective and acceptable intervention.

In summary, the results of our literature review showed that although CBT-gsh is a promising treatment for college students, there is substantial room for new modalities of delivery and content integration. The limitations of past CBT-gsh studies included high attrition rates (particularly when there was no or low therapist involvement), few adaptations designed for use in university student populations, a lack of gender representation and inclusivity, and treatment delivery of self-help content being delivered primarily through textbooks, instead via eHealth and mHealth platforms, which would provide increased opportunities for engagement and dissemination. With these considerations in mind, we chose to develop an mHealth app based on CBT-gsh, which would be paired with brief coaching sessions delivered either in person or via telehealth. We viewed the mHealth treatment delivery method as potentially helpful for reducing barriers to accessing ED care because students would not have to carry an additional book or read lengthy text descriptions and could increase engagement with the material through the provision of short quizzes and short-answer questions. Our goal was to create an mHealth app that would also seamlessly integrate meal logs and tracking tools within 1 digital tool. We chose to have shorter sessions (25-30 minutes) and a short duration of therapy (11 vs 20 weeks) because this would reduce waitlist time while likely not adversely affecting treatment efficacy. Finally, we viewed the overall concept of guided self-help as attractive to students because Tavolacci et al [11] found that students reported wanting to “try something on their own first,” which is compatible with a self-help model. To better serve a diverse university population, we wanted to create content that was gender- and size-inclusive and appealed to a wide range of students, including traditional college students (eg, students aged 18-24 years), nontraditional college students (eg, students aged >24 years and students with dependents), and graduate students. Improving mental health support for nontraditional students is particularly important, as these students are already at a higher risk for noncompletion of their degrees [31] and untreated mental health problems may worsen such outcomes.

### Phase 2: Intervention Development

#### Treatment Overview

The initial content of the app was decided over a 3-month period in weekly meetings between KTF and SRG. On the basis of the literature and our clinical experience, we created 14 brief modules that were designed to be delivered over an 11-or-12-week period with a built-in “buffer” week to account for the possibility of a missed or absent week. The modules covered topics from CBT-E, Cognitive Behavioral Therapy, and Dialectical Behavior Therapy. We designed modules to take 5 to 15 minutes to complete and focused on key information or lessons that were short and interactive. We embedded evidence-based routine outcome monitoring (ROM) within the app, given that research has shown that ROM results in increased end-of-treatment recovery rates, reduces patient deterioration for patients who show a poor initial response to treatment, and increases overall positive treatment outcomes at the end of treatment. Thus, BEST-U leveraged frequent ROM via surveys administered daily, weekly, and monthly to increase both client and therapist awareness and the targeting of problem behaviors. Clients reinforced their learning through weekly 25-to 30-minute telehealth “coaching” sessions with a trainee or licensed provider to review module material, solve problems, practice skills, and plan homework exercises. Trainees were graduate students currently enrolled in a clinical psychology doctorate program or postdoctoral fellows. Trainees were required to pass a graduate-level course focused on the treatment of EDs or the University of Oxford CBT-E training. All trainees were supervised by a licensed provider and attended weekly supervision meetings to review the cases. Throughout the app, we referred to the user’s trained therapist as a “Coach” because we found that participants found this term more empowering and less stigmatizing; therapists reported no concerns with the term “Coach,” given that it helped reduce barriers for clients seeking care.

#### Treatment Content

The treatment schedule is provided in Table 3. In the first half of the treatment, participants were provided psychoeducation about unhealthy eating behaviors and weight. Participants were provided with material to increase awareness of their thoughts, emotions, and behaviors and learn skills to reduce engagement in problematic eating behaviors, such as bingeing, purging, excessive exercise, and restriction. In the second session, the participants were introduced to regular eating and encouraged to engage in regular eating (ie, eating 3 meals and 3 snacks per day) throughout the program. Throughout the treatment period, the program identified targets for change and measured progress through frequent monitoring. Clients were asked to complete daily meal and ED behavior logs, weekly surveys on ED symptoms, and weekly self-weighing. The latter half of the treatment focused on reducing dysfunctional cognition and behaviors related to body image, overconcern with shape and weight, and body comparison. Strategies for change throughout the treatment included behavioral strategies (behavioral activation [ie, “disrupters”] and urge surfing), challenging cognitive distortions (Mindtraps), behavior chain analysis (BCA), interpersonal effectiveness skills, and exposure techniques (open weighing, food exposures, body exposures).

**Table 3:** Schedule of treatment content.

Week and module name		Module topics	Coaching session topics	Homework	Handouts or worksheets
**1**					
	*1. Starting Well*	Psychoeducation info about BEST-U^a^ structure	Orientation to treatment structure Goal setting Introduction to meal logs Review modules 1 and 2 Problem-solving barriers to weekly weighing	Begin weekly weighing	Participant expectations guide Weekly weighing handout
	*2. What’s Weight Got to Do With It?*	Psychoeducation about weight Rationale for weekly weighing			
			Orientation to treatment structure Goal setting Introduction to meal logs Review modules 1 and 2 Problem-solving barriers to weekly weighing		
**2**					
	*3. What are Unhelpful Habits?*	Psychoeducation about dieting and binge-eating behaviors	Review modules 3 and 4 Problem-solving barriers to regular eating Plan daily structure for regular eating	Begin engaging in regular eating	Binge cycles handout
	*4. Regular eating*	Rationale for regular eating	Review modules 3 and 4 Problem-solving barriers to regular eating Plan daily structure for regular eating		
**3**					
	*5. More Unhelpful Habits*	Psychoeducation about compensatory behaviors Overvaluation and self-comparison	Problem-solving regular eating challenges Review modules 5 and 6 Identify disruptors and urge surfing Reflection on treatment progress so far	Implement disruptors and urge surfing to reduce disordered eating	—^b^
	*6. The Disruptors*	Instruction on using behavioral disruptors and urge surfing to reduce engagement with unhelpful urges	Problem-solving regular eating challenges Review modules 5 and 6 Identify disruptors and urge surfing Reflection on treatment progress so far		—
**4**					
	*7. Mindtraps*	Identifying and challenging Mindtraps (ie, cognitive distortions)	Review module 6 Therapist guides the client in identifying and challenging a recent Mindtrap	Notice and challenge Mindtraps in daily life	Mindtraps worksheet
**5**					
	*8. Break the Chains*	Video instruction in using BCA^c^	Review module 7 Practice writing a BCA in session	Complete a new BCA	BCA worksheet
**6**					
	*9. Interpersonal Effectiveness*	DEAR MAN GIVE FAST interpersonal effectiveness skills	Review module 9 Practice formulating a request using DEAR MAN	Use DEAR MAN to make an interpersonal request	Interpersonal effectiveness worksheet
**7**					
	*10. Dieting*	Three aspects of eating (when to eat, how much to eat, what to eat) Dietary rules	Review module 10 Discuss client’s experience with dietary rules	Challenge a dietary rule (behavioral exposure)	—
**8**					
	*11. Body Image IQ*	Overconcern about shape or weight Information about comparisons	Review module 11 Discuss client’s body image concerns	Body comparison exercise	—
**9**					
	*12. Shape and Weight*	Body checking	Review module 12 Discuss the client’s experiences with body checking Complete values pie-chart activity	Complete 2 days of body-checking logs Engage in a valued activity identified from the pie chart	—
**10**					
	*13. Body Avoidance*	Body avoidance Body beliefs activity	Review module 13 Discuss the client’s experiences with body avoidance	Complete body exposure exercise	—
**11**					
	*14. The Road to Success*	Reflection on progress in BEST-U and plans for future	Review module 14 Formulate discharge plan Discuss lapses or relapses Goals for future Evaluate the need for additional treatment	Complete body exposure exercise	—

^a^BEST-U: Building Healthy Eating and Self-Esteem Together for University Students.

^b^No handout or worksheet included.

^c^BCA: behavior chain analysis.

#### App Overview, Content, and Structure

**Figure 3:**
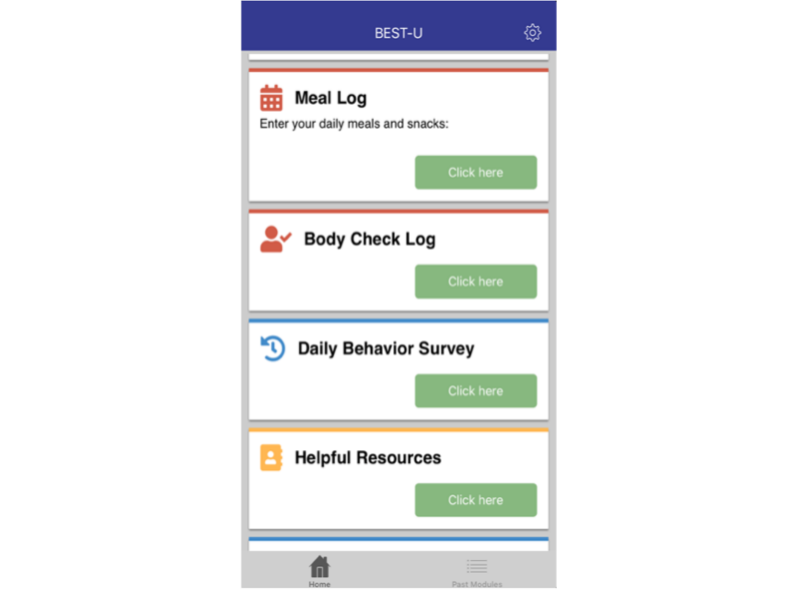
Home screen. BEST-U: Building Healthy Eating and Self-Esteem Together for University Students.

**Figure 4:**
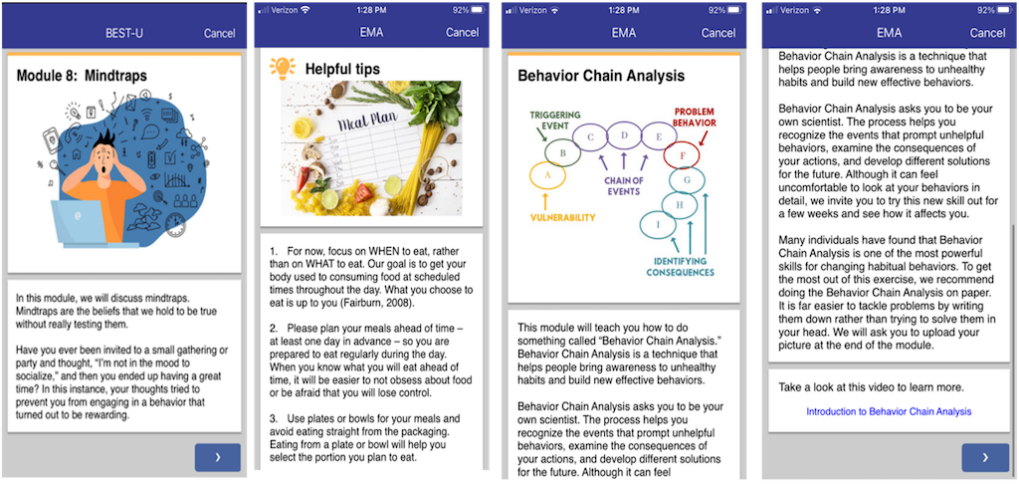
Sample Building Healthy Eating and Self-Esteem Together for University Students (BEST-U) module images. EMA: ecological momentary assessment.

The BEST-U home page consisted of 2 tabs: the home tab and the past modules tab (Figure 3). At the top of the home tab is a welcome message, followed by the action items. The action items included assessments assigned to be completed during a given week. Below the action items were the assessment items that the participants completed daily, including meal logs, body-checking logs, and daily behavior surveys. All assessments available on the home tab are described in further detail below. The last component of the homepage was the assigned module or modules for that week. There were 14 modules to be completed during the 11 weeks of treatment. For the first 3 weeks of treatment, the participants were asked to complete 2 modules per week. For the remaining 8 weeks of treatment, participants completed 1 module per week. Every module was built upon the information from the preceding module; therefore, we encouraged clients to complete them in the designated order. If a participant forgot to complete a module during the week it was assigned, they were asked to complete it the following week before beginning the newly assigned module. All modules from the previous weeks could be accessed and completed by navigating to the past modules tab on the main screen of the BEST-U app. Throughout the modules, we included images, inspirational quotes, and pictures to make the app attractive and engaging. To make the app as inclusive as possible, we selected images that showed a range of body sizes, races, ethnicities, and gender identities (see Figure 4 for sample images).

**Figure 5:**
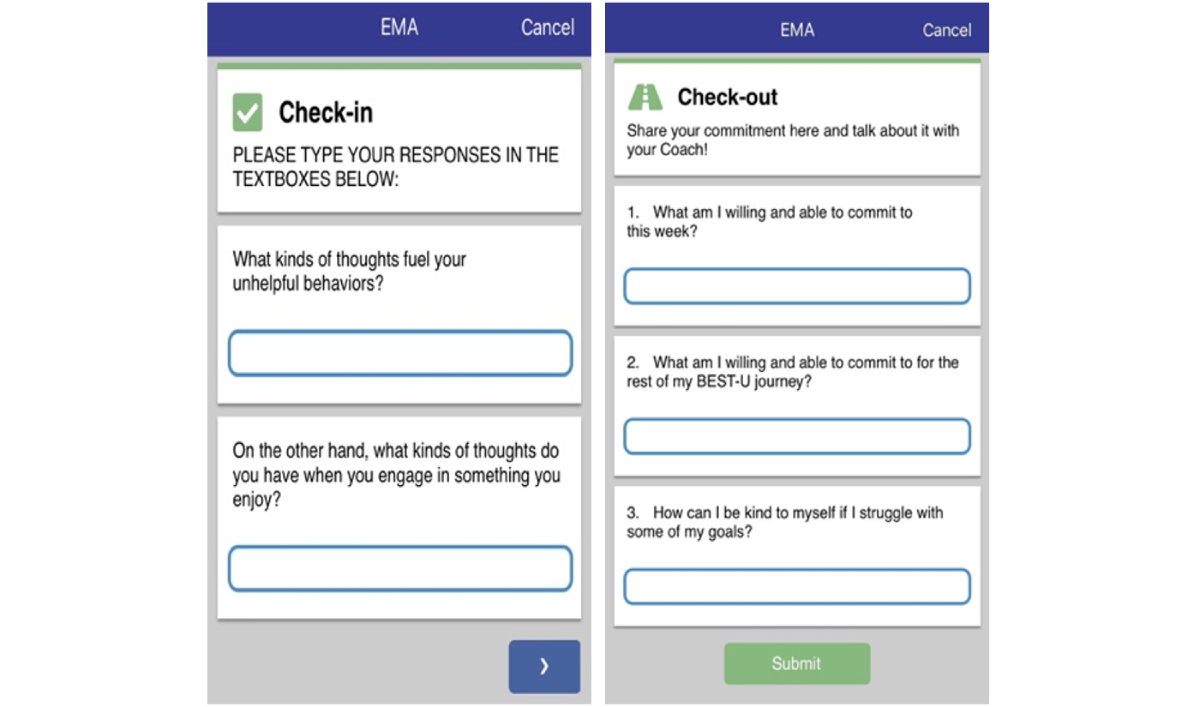
Check-in and check-out questions. EMA: ecological momentary assessment.

Each module began with a check-in question. These questions assessed issues, such as how well the participants were doing with the knowledge and skills gained from the previous week or module. The check-ins served as an informal introduction to the new module’s content by asking the participants to reflect on their experiences with a topic before receiving instructions on that particular topic. After the check-in (Figure 5), the app provided the instructional content. The instructional content included an introduction to the topic, a main lesson, and an interactive component. The interactive component was designed to deepen the participants’ connection to, and understanding of, the instructional content. These components included activities such as “Q&As” (eg, “What kinds of thoughts fuel your unhelpful behaviors?”), trivia, myth-busting (eg, “True or False? Counting calories is an effective form of weight control”), or behavioral exercises. The behavioral exercise from the Body Avoidance module, for example, asked participants to do 2 things they currently avoided due to body image concerns (eg, exercising in public and eating with other people). Each module concluded with a check-out question. Check-out questions provided an opportunity for the students to reflect on their treatment goals, barriers and solutions for change, motivations to change, and the quality of the participants’ relationships with their Coach. The questions also encouraged the participants to reflect on how to incorporate the instructional content into their lives. For example, the check-out questions from the Mindtraps module asked participants to consider a time when a Mindtrap (ie, a cognitive distortion) led to a negative consequence and to think about how they could challenge that Mindtrap if it happened again.

#### CBT Routine Outcomes Monitoring Within the App

ROM is critical because it allows coaches to monitor change (or a lack of change) on a regular basis and incorporate information from the assessments into weekly coaching sessions. Thus, BEST-U clients completed daily, weekly, and monthly assessments to track the outcomes of ED psychopathology and overall internalizing symptoms. The advantages of embedding assessments within the BEST-U app were the ability to track time to monitor adherence to the intervention and the ability of coaches to review the information before coaching sessions to improve treatment planning and session preparedness. Some assessments, including Meal Logs, Behavior Surveys, and Weight Logs, are part of the CBT monitoring for EDs.

#### Meal Logs

**Figure 6:**
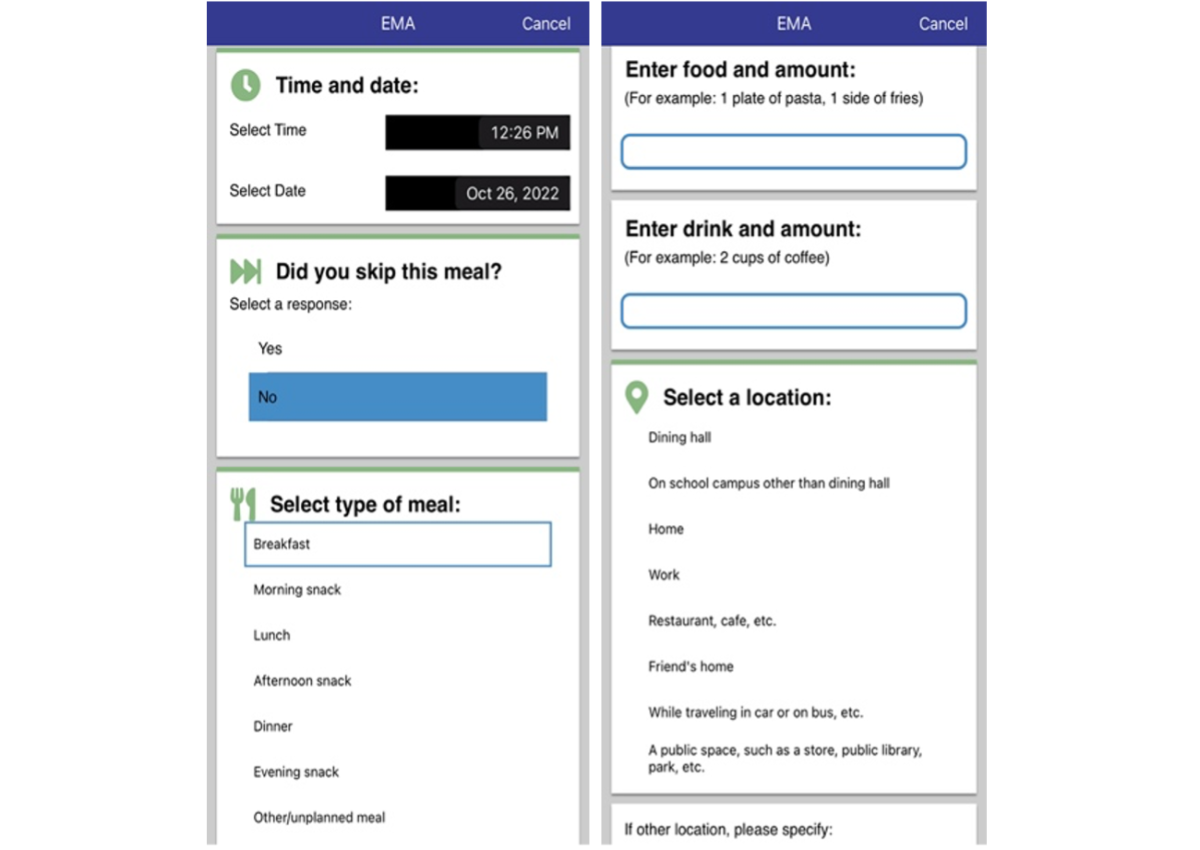
Meal log images. EMA: ecological momentary assessment.

The BEST-U clients completed daily meal logs electronically within the app (Figure 6). In each meal log, participants reported the time, date, and location (eg, home, dining hall, and restaurant) of each meal or snack consumed. After noting the type of meal (eg, breakfast, morning snack, lunch, afternoon snack, dinner, evening snack, or other or unplanned meal), the participants were asked to describe the type and amount of food consumed. In addition to information about the meal, meal logs included an assessment of emotional states and thoughts during the meal. Participants also reported whether they engaged in any ED behaviors (eg, binge eating, purging, self-harming, restricting, or excessive exercising) before, during, or after eating.

#### Behavior Survey

**Figure 7:**
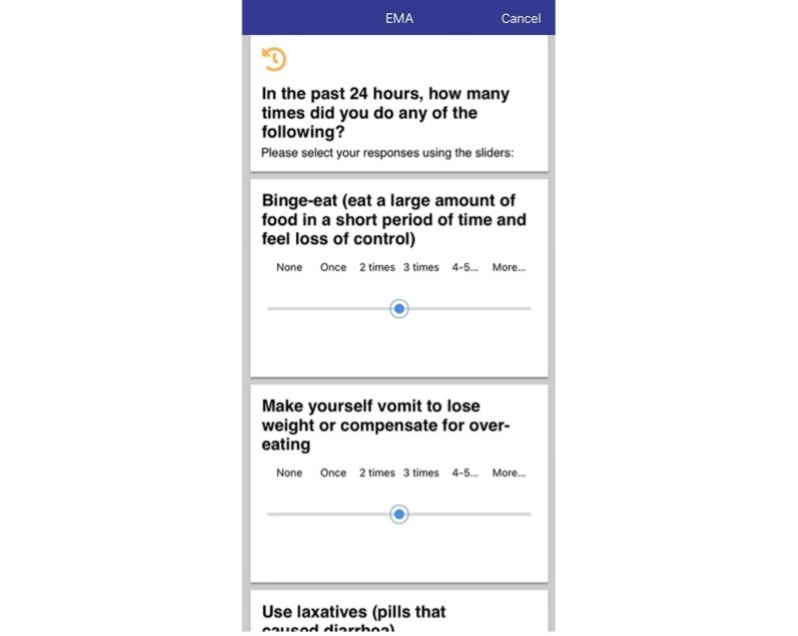
Sample image from the Daily Behavior Survey. EMA: ecological momentary assessment.

To monitor the frequency of specific ED symptoms and emotional states, BEST-U clients completed a daily behavioral survey (Figure 7). Participants reported how often they engaged in objective binge eating, self-induced vomiting, laxative and diuretic misuse, and other diet supplement use on a 6-point Likert scale ranging from “none” to “six or more times*.*” To assess dietary restriction, participants replied “yes” or “no” to questions about meal or snack skipping and restriction (eating <1200 calories per day). Body checking and body comparison surveys became available in the latter part of the treatment when the topics of body image concerns and overvaluation of weight and shape were introduced. When available, body checking and body comparison frequency were assessed using a 6-point Likert scale ranging from “none” to “50 or more times” in the past 24 hours. Finally, the frequency of positive and negative affect over the past 24 hours was assessed on a 5-point Likert scale ranging from “very slightly” to “extremely*.*”

#### Weight Log

**Figure 8:**
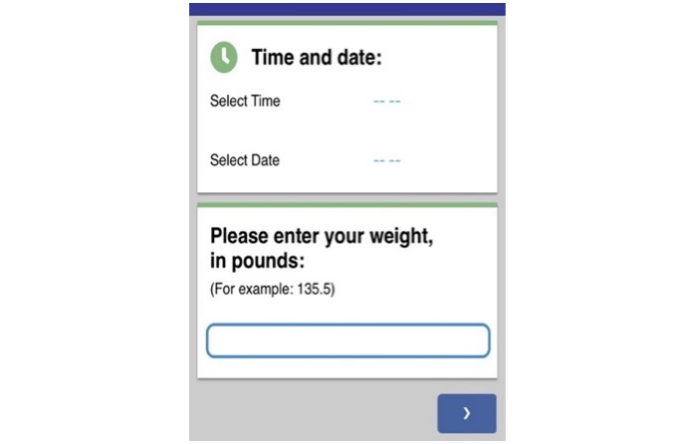
Weight log.

Weight logs (Figure 8) were completed to monitor weekly weighing when in-person weighing procedures were not possible (eg, clients who fully used telehealth). The participants were instructed to weigh themselves only once weekly, preferably on the same day and around the same time each week. Coaches reviewed potential barriers to weighing with clients at the first coaching session, including problem-solving access to a scale, if one was not available in their home (eg, borrowing a scale and weighing at the student health care center, gym, or a friend or family member’s home). Within the weight log, clients logged their weekly weight, and the date was automatically recorded. Clients also logged any emotions, thoughts, or ED behaviors that occurred in the context of weekly weighing. The weight log feature was included because weight exposure is a core CBT-E and CBT technique for increasing participants’ acceptance of their body by reducing avoidance and fear associated with the number on the scale [32,33].

#### Other Study-Related Outcomes Assessments

We included additional surveys in the app to assess the outcomes for our larger clinical trial and to enable clinicians to further monitor aspects of eating pathology and related forms of psychopathology during the treatment. These measures are described below.

#### Eating Pathology Clinical Outcomes Tracker

A weekly version of the Eating Pathology Symptoms Inventory (EPSI) was completed in the app to monitor 8 dimensions of ED psychopathology including body dissatisfaction, binge eating, restricting, cognitive restraint, excessive exercise, muscle building, and negative attitudes toward obesity. The EPSI [34] is a 45-item self-report questionnaire completed monthly to monitor changes in ED psychopathology. Participants respond to the 45 EPSI items on a 5-point Likert scale from 0 (“never”) to 4 (“very often”), with instructions to focus on the past week. In past research, the EPSI has shown evidence of a strong factor structure, excellent internal consistency (α=.84-.89), and test-retest reliability of scales over a 2- to 4-week time frame (*r*=0.61-0.85). The EPSI demonstrated evidence of good convergent validity when compared with other ED self-report measures and discriminant validity when compared with a measure of internalizing symptoms [34-36].

#### Positive and Negative Affect Schedule–Expanded Form

The Positive and Negative Affect Schedule–Expanded (PANAS-X) form [37] was completed monthly within the app to monitor changes in positive and negative affect. The PANAS-X is a 60-item self-report scale in which participants report the extent to which they have experienced different emotions over the past few weeks on a 5-point Likert scale ranging from 1 (very slightly or not at all”) to 5 (“extremely”). Past research on the PANAS-X has shown evidence of excellent internal consistency of positive affect (α=.83-.90) and negative affect (α=.79-.93) scales and convergent validity of positive and discriminant validity [37].

#### Inventory of Depression and Anxiety Symptoms

The Inventory of Depression and Anxiety Symptoms (IDAS-II) [38] was completed monthly in the app to monitor changes in the internalizing symptoms of anxiety and depression, including general depression, dysphoria, panic, social anxiety, insomnia, lassitude, ill-temper, traumatic intrusions, traumatic avoidance, well-being, suicidality, appetite loss, appetite gain, mania, euphoria, claustrophobia, checking, ordering, and cleaning. The IDAS-II was administered to assess monthly levels of anxiety and depression. Participants responded to 99 items related to anxiety and depressive symptoms over the past 2 weeks on a 5-point Likert scale ranging from 0 (“never”) to 4 (very often).” In past research [38-40], the IDAS-II scales demonstrated evidence of convergent validity with other measures of mood and anxiety (*r*=0.44-0.68), discriminant validity, and 2-week test-retest reliability (*r*=0.54-0.76).

#### Clinical Impairment Assessment

The Clinical Impairment Assessment (CIA) [41] is a 16-item self-report measure of psychiatric impairment secondary to an ED over the past 28 days. CIA was completed monthly within the app. The CIA was rated on a 4-point Likert scale ranging from 0 (not at all) to 3 (a lot).” The CIA demonstrated excellent internal consistency as measured by Cronbach α (α=.93) and item-total correlations (*r*=0.57-0.81) in past research [41,42]. The CIA also demonstrated acceptable test-retest reliability (intraclass coefficient 0.86) over 3 days and convergent validity with other measures of ED psychopathology in past studies (*r*=0.27-0.68) [41,42].

#### App Integration With Telehealth Coaching Sessions

##### Session Format

The BEST-U app was supplemented with 25 to 30 minutes of weekly telehealth coaching sessions by a trainee or licensed provider. Each coaching session was intended to adhere to the basic CBT session structure, which included agenda-setting, check-in, homework review, discussion of agenda items, and homework assignments. To increase compliance and consistency among coaches and to lower barriers for future dissemination, we developed a *Coach’s Manual*, which provided brief session outlines, required session activities, and a summary of information collected by the app to be reviewed before the session.

##### Coaching Session Activities

In most CBT-gsh programs, coaching sessions are used for problem-solving, motivation, and increasing treatment compliance. BEST-U coaching sessions were intended to accomplish these goals as well as to help the client apply the skills, strategies, and lessons from the modules to meet their own unique needs in the context of their personal circumstances. BEST-U therapists engaged in discussions and used handouts and worksheets that they shared on their computer screens to increase the client’s understanding of key CBT and Dialectical Behavior Therapy skills through structured activities. For example, clients worked with their coach to identify and challenge Mindtraps using structured worksheets, completed a practice BCA in session, and formulated effective interpersonal requests (DEAR MAN GIVE FAST skills from Dialectical Behavior Therapy).

BEST-U coaching sessions built upon material within the modules and extended them to the client’s specific eating and body image concerns. For example, clients and coaches designed individually tailored exposures related to dietary rules (eg, eating feared foods), body checking (eg, response prevention techniques), and body avoidance (eg, clothing or mirror exposure), which were then assigned for homework. Coaches and clients completed an exercise from CBT-E in which they created a pie chart to visualize the factors that determined the client’s self-worth. The pie chart helped clients visualize the amount of the “pie” taken up by overvaluation of weight and shape, which served as a discussion launcher for replacing overvaluation with activities that would increase self-esteem and value-directed living.

Coaching sessions also carried forward the skills and strategies learned in the previous modules to reinforce learning. For example, after cognitive change strategies were discussed in the app, clients were encouraged to identify Mindtraps when they arose in a session. Similarly, clients were encouraged to complete BCAs when problem behaviors occurred throughout the treatment. Clients were challenged to consider how they could use their new skills (eg, disrupters and urge surfing) when they described relevant situations in the review of homework or logs. Clients were taught that through BEST-U, they were learning skills and ways of thinking that could be applied to future challenges related to eating as well as general situations, such as life stressors, depressive symptoms, and anxiety symptoms.

The final coaching session focused on a review of treatment progress and the collaborative development of a written discharge plan. The purpose of this session was to help clients summarize their progress toward their goals, reflect on the skills learned, identify potential signs of relapse, and set new wellness goals for their personal development. Together with their coach, the clients identified “warning signs” for relapse and problem-solved strategies for managing relapses. Finally, we asked clients to identify their areas of desired future development and how they could work toward those with the skills they had learned through BEST-U. The participants were provided with a copy of their discharge plan to use as a guide after completing the program.

#### Postdischarge Check-Ins

After the discharge, BEST-U clients had the option of scheduling a 25- to 30-minute check-in session with their coach at the 3- and 6-month follow-up. These sessions involved a collaboratively set agenda, with no formal lessons or information provided. Sessions typically involved briefly reviewing the client’s progress over the treatment, discharge plan, and discussing any problem areas or challenges that the client experienced or anticipated would arise in the future.

#### Compliance and Engagement

We computed the participants’ survey compliance data based on 7 participants who completed the BEST-U 1.0 program and provided feedback. The compliance and outcome data from the other 8 participants in BEST-U 1.0 who participated in a multiple-baseline design trial are reported in a separate paper [21]. Engagement was assessed based on the number of modules that the participants submitted. Module submission requires that participants scroll through each screen of the module, answer prompts within the app, and click “submit” to submit their module as completed. As defined by module completion, the completion rate of the weekly modules was 89% (87/98), which indicated high engagement in BEST-U 1.0. The submission rate for the Weekly Weight logs was 68% (57/84). The submission rate of Weekly Surveys was 61% (51/84). Compliance with the Monthly Survey was 64% (9/14). The participants had extremely high compliance with the coaching sessions, with 100% of the sessions attended. In contrast, participants completed 32.7% (192/588) of the Daily Behavioral Surveys, which was lower than the ideal. Although not reported here, compliance and engagement data for the other 8 participants who participated in the multiple-baseline design study were similar to those reported for the other BEST-U 1.0 participants [21].

#### User Satisfaction and Usability

Table 4 provides the user satisfaction, usability, and attractiveness ratings from BEST-U 1.0 participants. The average scores were calculated for each question. The scores ranged from 1 (“strongly disagree”) to 7 (“strongly agree”). Mean scores of ≥5 (“somewhat agree”) were used as evidence for acceptability. Mean scores of ≥6 (“agree”) were used as evidence for “high” acceptability. Participants’ feedback on BEST-U as a treatment package was positive, with an acceptability grand mean of 5.89 across all quantitative survey items for BEST-U 1.0. The participants reported that they planned to continue using the resources, information, and activities learned in BEST-U to continue working on their goals. Participants also reported that they would recommend BEST-U to a friend experiencing similar problems. Participants were highly satisfied with the brief coaching sessions, as evidenced by the fact that all questions about coaching were rated ≥6 on average. Participants reported that they enjoyed the study, felt comfortable using the app, and that the amount of time spent reading the content of the modules was reasonable. In the qualitative survey, 5 participants endorsed items that indicated that the program had a positive impact on their body image. These data also corresponded to quantitative ratings of items indicating that they felt their eating behaviors had changed and were proud of the changes they made in the program. App attractiveness was the area of greatest concern, with an average rating of<5.

Qualitative comments from the participants indicated that they enjoyed the coaching sessions, felt they learned new coping mechanisms and skills, liked the overall structure of the program, and felt they improved their eating behaviors. One participant commented that the app was “very easy to navigate.” These positive qualitative comments were consistent with the participants’ quantitative ratings. Overall, both the quantitative and qualitative results showed that the BEST-U app was satisfactory and acceptable, including the aspect of ease of use and time spent on the app.

Students’ qualitative comments about changes that could be made to the app were largely focused on the attractiveness and availability of the meal logs. Student comments related to app attractiveness included the following:

Making the app more user friendly, and allowing answers to be saved, The overall view of the app, The app itself might need some more polish, and The app was not amazing, but it was not terrible. It could definitely have a better, more attractive layout. It is overall user-friendly but feels like it was made 15 years ago and could use some updating.

The other major area of concern was the lack of ability to have continuous access to meal logs. One student commented as follows:

I would have preferred access to my meal logs to just keep track for myself without having to log them elsewhere. It would also have helped me eliminate any confusion I may have had about entering meals with wrong times (frequently the AM and PM confusion after submitting the log).

**Table 4:** User satisfaction and feedback on the app^a^.

Question	Mean rating	
	BEST-U^b^ 1.0	BEST-U 2.0
1. I enjoyed participating in this study	5.94	6.02
2. I felt comfortable using the app.	5.73	5.75
3. If I needed to seek help again, I would come back to your program.	5.67	5.9
4. The smartphone app was easy to use.	5.40	5.37
5. The amount of time I spent answering questions on the app was reasonable.	5.14	5.27
6. The amount of time I spent reading modules on the app was reasonable.	5.93	6
7. I was satisfied with the amount of coaching I received.	6.47	6.16
8. The coaching I received helped me deal more effectively with my problems.	6.67	6.02
9. If a friend were in need of a similar help, I would recommend they try BEST-U.	6.47	6.12
10. The smartphone app was attractive.	4.07	4.19
11. It was easy to schedule times to talk with my Coach.	6.80	6.47
12. I changed my eating behaviors and habits.	6.14	5.61
13. I improved my body image.	5.13	5.18
14. I was happy with the length of the program.	5.73	5.68
15. I learned new information about health, eating, and body image.	6.20	6.06
16. I am proud of the changes I made during the BEST-U program.	6.27	5.8
17. I plan to use the resources, information, and activities to keep moving toward my goals.	6.40	5.96

^a^Acceptability grand mean for BEST-U 1.0: 5.89; acceptability grand mean for BEST-U 2.0: 5.73.

^b^BEST-U: Building Healthy Eating and Self-Esteem Together for University Students.

### Phase 3: Redesign

After the pilot study had concluded, the coaches participated in a *Coaches Workshop* to discuss results and share concerns and suggestions for the changes to be implemented in a second version of BEST-U. Coaches reported that several participants expressed interest in having continuous access to the information from meal logs and daily surveys. However, because PiLR does not easily allow these data to be saved within the app, coaches decided to instead suggest that interested participants keep a notebook with notes from modules, logs, and coaching sessions. Coaches also commented about their own usability of the PiLR data. With coaching sessions being brief, coaches explained that it was difficult to efficiently discuss meal logs with participants because the data were not neatly presentable and were overwhelming for participants (and coaches) to view. Coaches recommended the use of a PDF that compiled the data into a user-friendly format so that it could be downloaded from the Coaches’ Dashboard and viewed and discussed with participants with ease. Thus, we worked with PiLR to build a PDF to automatically integrate information from meal logs and daily behavior surveys into an easy-to-read format that coaches could download.

The coaches also raised concerns about a module that focused on alcohol use and peer pressure at college parties as a trigger for binge-eating episodes. We decided to remove the module because the coaches noted that many of the participants reported in the sessions that they did not drink alcohol, and nontraditional students often reported that college “party culture” was not relevant to their current problems. The coaches also noted that several participants reported interpersonal issues that contributed to the maintenance of unhealthy eating habits and poor body image (eg, unhelpful comments from family or friends about weight and shape), but they did not have the skills to address these issues. Thus, we replaced the alcohol and peer pressure module with an interpersonal skills module, which coaches thought would be more universally applicable and is in line with research on interpersonal dysfunction among people with EDs [43,44].

In addition to replacing the alcohol and peer pressure module with an interpersonal skills module, we made slight changes to the content and ordering of other modules. For example, in the initial version of BEST-U, a single coaching session was dedicated to the discussion of both BCA and Mindtraps. Coaches reported that there was too much content that week, and they were routinely unable to cover all material in 1 session. To allow more time for each of these complex topics, we split the content into 2 separate sessions. The coaches reported that the complexity of BCA was difficult to explain in a brief coaching session, even with the inclusion of introductory information provided in the app module. To address this concern, we created and embedded 2 engaging videos in the BCA module that clearly defined the rationale for BCA and provided an illustrative example of how BCA can be used to change problematic eating behaviors and body image concerns to allow participants to gain an understanding of the skill before attending their coaching session.

Coaches discussed the variability regarding the completion of surveys in the app. Specifically, the coaches expressed concern that the number of surveys participants were asked to complete was potentially burdensome and could discourage participants from engaging in the program. The team considered eliminating the weight log and weekly survey to reduce the assessment burden, but the surveys were ultimately kept to ensure that we had ROM and because weight exposure is a core CBT-E technique for EDs. Participants’ access to the treatment modules was extended to 6 months after discharge, which was better aligned with their total time enrolled in the study.

Finally, in terms of app attractiveness, we had limited ability to update the “look” of the app because the PiLR platform functions similarly to web survey building tools, such as Qualtrics, and the layout and format is subject to their overall platform design. Although we added videos that our team felt were highly attractive and additional images to the app to improve the attractiveness, we were limited in our ability to fully address this concern.

### Phase 4: Further Pilot-Testing of Usability and Acceptability

#### Compliance and Engagement

Survey compliance data were based on 48 participants who completed the 11-week BEST-U 2.0 program and provided feedback. Engagement was defined as the number of modules submitted per week. Participants submitted 90.3% (607/672) of the weekly modules, indicating high engagement with BEST-U 2.0. Compliance with the Weekly Survey was 64.4% (371/576), and approximately 0.03% (6/18185) of the data from the submitted Weekly Surveys were missing. Missing Weekly Surveys were largely owing to system errors within PiLR, which led to a small number of surveys not being displayed on participants’ home screens. Most system errors occurred within a 2-week period during which PiLR released a system update on iOS. During this time, the participants who were impacted were sent paper-based surveys. Compliance with the Weekly Weight Log was 71%. (409/576). The Monthly Survey compliance was 73% (70/96), and 0.32% (41/13003) data from the submitted surveys were missing. Finally, participant compliance with the Daily Behavior Survey, which assessed engagement in ED behaviors each day was relatively low with average compliance of 32.66% (1317/4032). In all submitted daily behavior surveys, 8.12% (2052/25266) of data were missing. Missing data from the weekly and monthly surveys were due to occasional bad Wi-Fi connections, which led to some responses not being recorded. Missing data in the daily behavior surveys were due to participants not finishing the survey or skipping the questions. Overall, compliance and engagement were higher in BEST-U 2.0 compared with BEST-U 1.0.

#### App Usability

The participants completed an ad hoc survey to assess their experiences using the BEST-U mHealth app and with their coach. The participants reported their level of agreement on a Likert scale ranging from 1 (“strongly disagree”) to 7 (“strongly agree.”). Table 4 shows the average ratings from BEST-U 2.0 participants (right column) in comparison with BEST-U 1.0 participants (left column). Mean scores of ≥5 (“somewhat agree”) were used as evidence for acceptability. Mean scores of ≥6 (“agree”) were used as evidence for “high” acceptability. BEST-U 2.0 had an acceptability grand mean of 5.73, indicating that the treatment was viewed as acceptable. The results showed that the highest-rated items reflected participants’ positive experiences with their coach and the program as a whole. For example, participants reported that they enjoyed participating in the study and would recommend BEST-U to a friend who was in need of similar help. Participants felt comfortable using the app and thought that it was easy to use. Participants reported that the amount of time spent reading modules and answering questions within modules was appropriate. Importantly, the participants were proud of the changes they made because of the program and thought that their body image and eating behaviors had improved; indeed, most participants planned to use the information they learned in BEST-U 2.0 to in the future. In terms of areas of relative weakness, similar to the initial pilot, the participants’ lowest rating was for app attractiveness.

In terms of qualitative feedback, similar to BEST-U 1.0, participants had positive comments about their experience with their coach and the overall structure of the program (eg, participants liked the combined app and coaching approach), and some participants specifically mentioned benefiting from the Mindtraps, BCA, and interpersonal effectiveness modules. Areas for improvement were consistent with the feedback reported in the quantitative survey and focused on (1) wanting to have a back button or other means for viewing meal logs once they were submitted and (2) improving the aesthetics of the app. One participant noted as follows:

The clunkiness and aesthetics of the app could be improved, but it was very functional...Being able to review and revise past food entries would be helpful. Sometimes I would get from page one to page two to log a meal that occurred earlier in the day and have realized I made a mistake and would have to start the log over or submit another log. It also would have been helpful to be able to see what I logged over time so I could have reflected on patterns for things like the BCA activity with that information because I did not always remember what I was feeling.

## Discussion

### Principal Findings

This paper describes the development, usability, and acceptability of the BEST-U mHealth app, which is designed to fill critical gaps in access to ED treatment on college campuses. Consistent with the recommendations for mHealth development [45], we used a 4-stage user-centered design process. The four phases included (1) needs assessment, (2) prototype development and initial evaluation in a pilot trial, (3) redesign, and (4) further pilot-testing to assess the usability and acceptability of the final version of the mHealth app. The aim of this approach was to create, deliver, and refine content that was acceptable, engaging, and efficacious for our end users.

The results from our provider-based survey indicated a high demand for services but a lack of available ED treatment resources for university students on our campus. These survey findings led us to investigate the feasibility of creating and implementing a new CBT-gsh mHealth app for university students to address the high demand for services by using a scalable tool with a relatively low provider burden. The BEST-U mHealth prototype was an 11-week program that provided interactive, weekly “modules” that focused on second and third-wave cognitive behavioral skills. BEST-U was paired with brief 25 to 30 minutes of weekly telehealth “coaching” sessions provided by a trainee or licensed provider.

To increase inclusion and challenge stereotypes that lead to stigma, we carefully considered age, weight, and race and ethnicity when we selected images and examples within the app and attempted to address concerns that our team perceived as common among university students. However, upon initial pilot-testing, we found issues with one of the app modules that therapists reported had low relevance to many of their client’s experiences. Specifically, the coaches raised concerns with a module that focused on alcohol use and peer pressure at parties as a trigger for binge-eating episodes. We decided to remove the module because coaches noted that many of our participants reported that they did not drink alcohol, and several nontraditional and graduate students reported that student “party culture” was not relevant to their current problems. Coaches also noted that several participants reported interpersonal issues that contributed to the maintenance of unhealthy eating habits and poor body image (eg, unhelpful comments from family or friends about weight and shape), but they did not have the skills to address these issues. Coaches expressed concerns about the organization of the app content, in which 2 of the most complex topics (Mindtraps and BCA) were covered in the same week, which led to therapists not being able to cover all topics that week. These issues were addressed in the redesign phase through the removal, addition, and reorganization of content in BEST-U, with the help of BEST-U therapists across 2 workshops. Finally, one of the more complex topics, BCA, was challenging to fully explain within the app, so we added 2 engaging videos to better explain BCA to students using realistic examples.

The results indicated that the revised version of BEST-U was highly acceptable and user-friendly. For example, participants thought that the overall treatment length and amount of time spent reading and answering questions in the app was highly acceptable. Participants liked the overall structure of BEST-U and particularly enjoyed the ability to briefly meet one-on-one with their coach each week. In the open-ended qualitative survey responses to the question, “What were the best things about the BEST-U program?” the participants overwhelmingly indicated that coaching was viewed as the most beneficial aspect of the program. For example, 31 participants indicated that coaching was a beneficial aspect of BEST-U; for example, one participant indicated, *Having a Coach to talk to and support me during this experience was really valuable.* Participants also felt that they benefited from the skills taught through the app, particularly the Mindtraps, BCA, and interpersonal effectiveness skills. In all, 18 responses focused on benefits related to the skills taught through the app; when asked, “What were the best things about the BEST-U program?” one individual reported *The modules about how our thoughts affect our actions (Mind Traps, Disruptors) and the weekly coaching*. Finally, because of the program, participants felt that they had improved their eating habits and body image. A total of 5 responses explicitly indicated improvements in body image, and 6 responses explicitly indicated improvements in eating habits, suggesting that BEST-U was perceived as providing a positive impact on their body image.

### Limitations

Our use of an mHealth app resulted in an intervention with many strengths; however, there were also challenges during the initial development and implementation of BEST-U. First, although we conducted a careful literature review as part of our needs assessment, we did not conduct a systematic review. Thus, we did not track metrics such as search terms used, number of articles accessed, or databases used in a systematic way. We also did not conduct separate focus groups of students or collect qualitative data on care providers’ perceptions of needs in phase 1 testing. Second, in terms of our study design, although we sent screens to all enrolled students, which increased our representation of underrepresented students within the study, the university as a whole had lower racial and ethnic diversity; consequently, most participants identified as White and cisgender women. However, the percentage of cisgender men participating (13/87, 15%) was consistent with the overall percentage of those with EDs who are male in our population. In addition, the percentage of participants identifying as a minoritized ethnicity (approximately 20/87, 23%) was lower than the percentage of students at the university identifying as a minoritized ethnicity (57%), indicating that work remains to be done to effectively reach these populations. Although we had more students in sexual or gender minority groups and more males compared with many past CBT-gsh studies on EDs [16,23], future studies are needed to expand and test the reach of BEST-U beyond a single university and to a broader range of diverse students. Third, we had a low enrollment rate relative to the number of students we screened. This issue was due, in part, to lower interest in participation at the beginning of the pandemic when several eligible participants decided not to participate because they were concerned about contracting COVID-19 at the in-person medical evaluation appointment. Toward the latter part of the project, we had a higher response than anticipated, with many more students expressing interest than we were able to serve, resulting in a staffing-driven bottleneck in admitting students to the program. This increased interest in participating may have been because of the sharp increase in EDs among university students since the start of the pandemic [46], which further increased students’ needs for ED treatment.

Fourth, we experienced a small number of technical problems. Our mHealth protocol required deviation from the standard use of PiLR, the platform upon which BEST-U was built. For example, we used longer assessment timeframes and data collection periods than those used in standard ecological momentary assessment (EMA) studies, which is the type of study for which PiLR is designed. For example, a recent review found that the average EMA study lasts for 7 days, with an average of 6 assessments per day [47]. The users of our program were instructed to log each meal and snack (recommended 3 meals and 2-3 snacks per day) for 11 weeks, in addition to a daily ED behavior survey; thus, our assessment schedule was equally intense on a daily level, yet longer than the typical study. At the same time, in traditional CBT-E, clients are asked to maintain a daily paper-and-pencil log for 16-21 weeks. These daily logs include foods eaten, whether an ED behavior occurred (eg, purging or binge eating), and any notes or observations the client may have. Thus, although the standard EMA period is much shorter than that in this study, the record for traditional CBT-E is longer and more burdensome. Our unique use of PiLR resulted in a small number of surveys not being delivered to the participants’ BEST-U app during the intervention. When technical issues arose, PiLR computer engineers thoroughly investigated and troubleshooted the issue, which required a temporary halt in access to the app. However, because the participants were actively engaged in our treatment when these issues arose, we were unable to pause the study, which limited the testing capabilities of the computer engineers. When the survey delivery malfunctioned, we emailed PDF versions of the weekly or monthly surveys to the participants. Completed surveys were emailed back to us and hand-scored by study team members. Eleven weekly or monthly surveys were completed in PDF format owing to the survey delivery malfunction.

Fifth, despite our redesign efforts, there were certain limitations of the BEST-U app. For example, the PiLR platform does not allow users to review submitted information, and once a user advances the content of the app, they cannot return to the earlier screens. The app is designed to immediately transfer all data from the app to PiLR’s HIPAA-compliant server to ensure the privacy of identifiable and personal information, particularly in the case of the user’s phone being stolen or accessed by another individual without consent. However, in certain situations, it could be useful for clients to be able to review previous logs on their mobile devices to identify patterns and changes in their moods and behaviors over time. For example, clients reported that they would like to have had the ability to access the meal logs submitted through the app. Providing clients with the web interface data in a session was not feasible because the data required understanding and skill of reading raw data from a relatively large and complex data set. Even Coaches, all of whom had training in data analysis and survey design, reported difficulty in quickly reading and interpreting the raw data from the server. We attempted to address issues related to the coach’s concerns about the readability of data by creating automated PDF weekly summary sheets for each log type. These summary sheets collated information from the raw data and provided an easier-to-read overview of data submitted through logs that could be viewed by coaches and shared with clients in a session. However, flaws in the summary sheets (eg, meal log information was not listed in chronological order) must be further improved in future versions of BEST-U for optimal clarity and usability.

Sixth, participants in BEST-U 1.0 reported concerns about app attractiveness. Although we added images and videos to BEST-U 2.0, we were limited in our ability to improve the attractiveness of the app because the PiLR platform has settings that limit the fonts, colors, and format options. Participants in BEST-U 2.0 indicated that app attractiveness was still an area of improvement. Finally, our team used a HIPAA-secure video platform, Healthie, to schedule meetings, conduct video calls, and document clinical notes. Ideally, we would have been able to conduct therapy within the BEST-U app, but embedding video call capabilities within the app was beyond our scope of funding and expertise. However, the advantage of having notes and video calls outside of BEST-U is that therapists in future research will have a broad range of options and can conduct coaching sessions on another platform or in person.

### Strengths

There are a number of strengths of BEST-U. First, we selected images and examples in an inclusive manner to encompass multiple types of students who are not traditionally considered in the development of treatment of EDs by depicting individuals with minoritized races and ethnicities and individuals with a range of body sizes and shapes. Second, individuals with EDs are often unable to identify whether their behavior is “serious” enough to warrant professional treatment. This distorted perception of need represents a significant barrier to treatment seeking [48]. To reduce this barrier, the term *coach* was used throughout the BEST-U app to refer to the clinician because *coach* had a more benign connotation than *therapist* and empowered the student as the one in charge of their progress. We conducted a survey of BEST-U providers, and none of them had concerns with the use of the term *coach* because they viewed it as reducing barriers to accessing care.

Third, the BEST-U intervention offered a great deal of autonomy and flexibility. Participants were able to complete their work on their own through the mHealth app, and the coaching sessions were offered via telehealth. Participants could engage in the intervention wherever they had internet access, which was helpful considering the variable schedules of the students. Because of this built-in flexibility, BEST-U was well suited to accommodate the numerous and frequent changes brought about by the COVID-19 pandemic (eg, university policies, living arrangements of students, and mandatory isolation following COVID-19 exposure). Fourth, BEST-U was able to send automated reminders to participants to complete meal logs and daily behavior logs throughout the day. Automated reminders were helpful in prompting clients to complete various logs, which may have improved treatment compliance. Fifth, the coaches had digital access to module completion, meal, and behavior logs through the web-based dashboard. This afforded the clinician the opportunity to more readily identify trends in the patient’s symptoms and treatment progress. Having digital access to this information before the session allowed coaches to plan appropriately, maximize their time with the patient, and reduce the time to enter and monitor data. Access to client data before the coaching sessions allowed a personalized approach that likely supported client engagement and allowed BEST-U skills to be practiced with personally relevant situations and concerns. Finally, the interactive nature of the app may have facilitated material retention, given that hands-on, active learning improves both short- and long-term memory than the learning methods that only include reading and lectures [49].

### Conclusions

University students are a group that is at risk of developing EDs, yet there are several barriers to accessing quality and evidence-based treatment. Digital interventions can address some of the barriers to treatment by facilitating access through providing flexible scheduling and eliminating transportation needs. However, mHealth remains an underused mental health intervention, particularly for EDs. Our guided self-help cognitive behavioral intervention, BEST-U, was designed to fill this intervention gap by offering a brief, scalable mHealth intervention for a diverse range of college students. Using an mHealth-supported intervention, which is a digital format that is familiar to a diverse range of college students [50], providing a private and confidential mechanism to record and store reports of thoughts and behaviors, shortening treatment length and session times, and incorporating interactive components, CBT-gsh through BEST-U is a promising new intervention with the potential to target ED symptoms in college students at scale.

## Abbreviations

BCA: behavior chain analysis
BEST-U: Building Healthy Eating and Self-Esteem Together for University Students
CBT: Cognitive Behavioral Therapy
CBT-E: Cognitive Behavioral Therapy–Enhanced
CBT-gsh: Guided Self-Help Cognitive Behavioral Therapy
CIA: Clinical Impairment Assessment
CONSORT: Consolidated Standards of Reporting Trials
ED: eating disorder
EMA: ecological momentary assessment
EPSI: Eating Pathology Symptoms Inventory
HIPAA: Health Insurance Portability and Accountability Act
IDAS-II: Inventory of Depression and Anxiety Symptoms
mHealth: mobile health
PANAS-X: Positive and Negative Affect Schedule–Expanded
REDCap: Research Electronic Data Capture
ROM: routine outcome monitoring
SB-ED: Student Bodies–Eating Disorder

## Data Availability

Descriptive-level data such as means, SDs, and correlation matrices are available for secondary analysis and meta-analysis upon request.
